# Predicting the Most Deleterious Missense Nonsynonymous Single-Nucleotide Polymorphisms of Hennekam Syndrome-Causing CCBE1 Gene, In Silico Analysis

**DOI:** 10.1155/2021/6642626

**Published:** 2021-06-10

**Authors:** Khyber Shinwari, Liu Guojun, Svetlana S. Deryabina, Mikhail A. Bolkov, Irina A. Tuzankina, Valery A. Chereshnev

**Affiliations:** ^1^Department of Immunochemistry, Institute of Chemical Engineering, Ural Federal University, Yekaterinburg, Russia; ^2^School of Life Science and Technology, Inner Mongolia University of Science and Technology, Baotou 014010, China; ^3^Medical Center Healthcare of Mother and Child, Yekaterinburg, Russia; ^4^Institute of Immunology and Physiology of the Ural Branch of the Russian Academy of Sciences, Yekaterinburg, Russia

## Abstract

Hennekam lymphangiectasia-lymphedema syndrome has been linked to single-nucleotide polymorphisms in the CCBE1 (collagen and calcium-binding EGF domains 1) gene. Several bioinformatics methods were used to find the most dangerous nsSNPs that could affect CCBE1 structure and function. Using state-of-the-art in silico tools, this study examined the most pathogenic nonsynonymous single-nucleotide polymorphisms (nsSNPs) that disrupt the CCBE1 protein and extracellular matrix remodeling and migration. Our results indicate that seven nsSNPs, rs115982879, rs149792489, rs374941368, rs121908254, rs149531418, rs121908251, and rs372499913, are deleterious in the CCBE1 gene, four (G330E, C102S, C174R, and G107D) of which are the highly deleterious, two of them (G330E and G107D) have never been seen reported in the context of Hennekam syndrome. Twelve missense SNPs, rs199902030, rs267605221, rs37517418, rs80008675, rs116596858, rs116675104, rs121908252, rs147974432, rs147681552, rs192224843, rs139059968, and rs148498685, are found to revert into stop codons. Structural homology-based methods and sequence homology-based tools revealed that 8.8% of the nsSNPs are pathogenic. SIFT, PolyPhen2, M-CAP, CADD, FATHMM-MKL, DANN, PANTHER, Mutation Taster, LRT, and SNAP2 had a significant score for identifying deleterious nsSNPs. The importance of rs374941368 and rs200149541 in the prediction of post-translation changes was highlighted because it impacts a possible phosphorylation site. Gene-gene interactions revealed CCBE1's association with other genes, showing its role in a number of pathways and coexpressions. The top 16 deleterious nsSNPs found in this research should be investigated further in the future while researching diseases caused CCBE1 gene specifically HS. The FT web server predicted amino acid residues involved in the ligand-binding site of the CCBE1 protein, and two of the substitutions (R167W and T153N) were found to be involved. These highly deleterious nsSNPs can be used as marker pathogenic variants in the mutational diagnosis of the HS syndrome, and this research also offers potential insights that will aid in the development of precision medicines. CCBE1 proteins from Hennekam syndrome patients should be tested in animal models for this purpose.

## 1. Introduction

Lymphangiogenesis is a process that helps the lymphatic system in its development. This includes migrations, proliferation, and budding of endothelial lymphatic progenitor cell lines [1–3]. The interstitial fluids, which are normally stored in the cardiovascular system, frequently flow away due to irregular Lymphangiogenesis, and this drainage can cause chylothorax, pleural effusion, angiectasias, lymphedema, and chylous ascites of lymph vessels in various organs, including the intestines [4]. Dysplasias's symptoms of lymph vessels are usually reserved for the limbs [1]. Hennekam syndrome is a genetically heterogeneous condition. Hennekam lymphangiectasia is a condition marked by disorders of the lymphatic system, which affects a variety of organs and links the gastrointestinal tract and the pericardium. Lymphedema demonstrates abnormal facial dysmorphism and cognitive dysfunction [5]. Approximately, up to now 45 people have been diagnosed with HS syndrome [6]. Almost 25% of patient's diseases are influenced by biallelic mutations in CCBE1 (Hennekam lymphangiectasia-lymphedema syndrome 1 (HKLLS1; MIM: 235510)) and FAT4 (Hennekam lymphangiectasia-lymphedema syndrome 2 (HKLLS2; MIM: 616006)) while CCBE 1 gene mutation [7]. In the examination of two siblings with missense, the type was found the biallelic mutation in the ADAMTS3 gene [8]. In humans and model organisms, the signaling protein collagen- and calcium-binding domain 1 (CCBE1) is required for lymphangiogenesis. As per forward genetic screening in zebrafish for a causative coding mutation in CCBE1, there is a mutant known as full of fluid (fof) that misses the thoracic duct's truncal lymphatic vessels but retains normal blood vasculature [9]. Missense mutation in the CCBE1 gene in the protein functional domain or upstream cysteine-rich domain of EGF was identified as the causative agent of HKLLS1 [6]. The CCBE1 gene plays a significant role in the growth of the lymphatic system in a model organism [9, 10]. However, the connection between FAT4 and lymphatic development is still not clear. Over time, our understanding of the phenotype associated with the CCBE1 mutation evolves. In the original account, the key inconsistency in the degree of cognitive damage (expansion from normal to moderate damage) is displayed by Hennekam syndrome subjects [11]. Specimens with clinically diagnosed Hennekam syndrome with or without mutations in CCBE1 were compared in the most recent study [6]. The CCBE1 gene interacts with connective tissue in the extracellular matrix and is then secreted [10–12]. Zebrafish often lacks lymphatic vessels and thoracic ducts, as well as the ability to develop edema [9, 11]. A mutation in the CCBE1 gene confirmed this. The same case of developing edema was shown in mice models [10]. On this basis, a mutation in this gene, which is thought to be the key gene between organisms, was linked to vascular lymphatic system dysfunction, leading to the conclusion that the human CCBE1 mutation is linked to widespread lymphatic dysplasia. Aagenaes syndrome, a rare AR condition, has also been linked to the biallelic CCBE1 mutation. This rare condition causes neonatal intrahepatic cholestasis, extreme chronic lymphedema without mental retardation, and lymphangiectasia [13]. Aagenaes syndrome was common in untreated children, and fetal hydrops was also found in HS patients [13, 14]. The proof that disease is caused by the rarity of a mutated allele is supported by the CCBE1 gene triggering the mutation in the latest evidence. Because of their segregation of phenotype in an AR inheritance model, their sporadic repetition in unrelated organisms, and the large number of associated carrying mutations, these mutated alleles may have a harmful impact [15]. Molecular biology, statistics, mathematics, computer science, and genetics all fall under the umbrella of bioinformatics [16]. Single-nucleotide polymorphism is the most common genetic variation present in the general population (SNPs). Every single nucleotide in the entire genome has been modified by SNPs. There are 200–300 bp SNPs in the human genome, but there are 5000,000 SNPs in the entire human genome. This can result in a variety of sequence changes, which can contribute to abnormal function [17–19]. Aside from SNPs in the exonic region of the genome, nonsynonymous SNPs (ns SNPs) and amino acid sequence changes in gene products are often affected by genetic variation (ns SNPs). SNPs do not have a large biological impact, but they can disclose a variety of disorders, such as affecting immunological response to drugs, and in some cases, SNPs can be used as biomarkers for disease vulnerability [20]. Changes in amino acid sequence caused by SNPs are responsible for 50% of reported cases of inheritance disorders [21]. Gene expression and transcription factor binding are also affected by promoter regions and regions outside of the gene [22, 23]. Single-nucleotide polymorphisms have a critical role to play in determining an individual's susceptibility to various diseases and drug reactions (SNPs). SNPs that cause disorders are discovered biologically through a simple procedure, so it is critical that we find them before they are used as a tool in genetics technologies [24]. Alignment methods based on matrix and data tree structure computation are used in the tools. Recent results, such as [25, 26], show that hash-based functions can speed up the entire process. The aim of this study is to use a variety of in silico approaches based on different concepts to investigate the potentially harmful effects of nsSNPs in the CCBE1 gene and protein. The study's aim is to provide a valuable tool for quick and cost-effective screening for pathologic nsSNPs, rather than biological experiment validation.

## 2. Methods

### 2.1. SNP Retrieval

Entrez Gene on the website of the National Center for Biological Information (NCBI) was collected from the data of the human CCBE1 gene. The information of SNP (protein accession number and SNP ID) of the CCBE1 gene was gained from NCBI dbSNP (http://ncbi.nlm.nih.gov/snp/) and SwissProt databases (http://expasy.org./). There was also searched other databases as Exome Aggregation Consortium, Genome Variation Server, and F-SNP to cross-check the nonsynonymous SNP (nsSNP) data for the CCBE1 gene [27]. The databases were accessed: 3 July 2020.

### 2.2. GeneMANIA

To check the interaction of the CCBE1 gene and observation of its association with other genes in order to predict the effect of nsSNPs on other related genes was used, GeneMANIA (https://genemania.org/) and STRING (https://string-db.org/cgi/) (accessed on 6 July 2020 using manual search for CCBE1 in the search box) [28]. Prediction of gene-gene interaction by GeneMANIA is that interaction is based on the basis of pathways, colocalization, coexpression protein domain similarity, genetic, and protein interaction. Predictions of STRING were limited to the top 10 best interactive genes with parameters that included gene fusion, co-occurrence, coexpression, and experimental and biochemical data. Those data showed a combined score for each gene's interaction with the target gene in range from 0 to 1, when 0 was the lowest interaction and 1 was the highest interaction. Therefore, CCBE1 was presented as our input gene and that generated the gene-gene interaction network.

### 2.3. Prediction Tool Used for nsSNP

#### 2.3.1. Sequence Homology Tool (SIFT)

For every sequences of query, the SIFT takes referential SNP ID and sequence of query by using multiple closely related information to prediction of tolerated and damaging substitutions [29, 30]. It tells whether the substitution is tolerated at that position. The tool was used on 6 July 2020.

#### 2.3.2. PolyPhen

(http://genetics.bwh.harvard.edu/pph2/) PolyPhen predicts by using specific empiric rules the effect of amino acids substitution on the protein's structure and function. Protein sequence, amino acid position, database ID/accession number, and amino acid variant details are the input for the PolyPhen [31], and the score difference between variants and wild-type amino acid is calculated. The tool was used on 6 July 2020.

#### 2.3.3. Analysis and Identification of the Most Damaging SNPs

Many algorithms for prediction of functional impact confirmed nonsynonymous single-nucleotide polymorphisms (nsSNPs). Those algorithms are SIFT [29, 30], PolyPhen2 [31], PROVEAN [32], M-CAP, LRT, META SVM, MetaLR, FATHMM-pred, FATHMM-MKL-coding-pred, Mutation Assessor, VESST3 CAAD, DANN, Mutation Taster by VarCARD [33], SNP-GO, PhD-SNP and PANTHER [34, 35], and SNAP2 [36]. These tools were used from 8 to 25 July 2020.

### 2.4. Prediction of Disease-Related Amino Acid Substitution and Phenotypes by MutPred

The online server MutPred (http://mutpred.mutdb.org/) is used as searching tool for prediction of the molecular basis of the disease which is related with amino acid substitution in a mutant protein [37]. It uses several attributes that are related to protein structure, function, and evolution. There are used three servers, PSI-BLASAT, SIFT, and Pfam profiles, along with TMHMM, MARCOIL, and DisProt algorithms. These are the prediction of some structural damages. The greater accuracy of prediction is reached by combining of the scores of all those three servers.

### 2.5. Prediction of Stability of the Mutated Protein due to SNPs by iStable 2.0

Amino acid substitutions are caused by missense SNPs and can change the stability of native protein which can lead to influencing of protein and in the end lead to diseases [38]. By a metaclassifier, iStable 2.0, we are predicting changes due to missense SNPs in protein stability. This metaclassifier uses machine learning and investigates the increasing or decreasing stability of the protein. It happens due to an amino acid substitution which is based on prediction of 8 structural-based (I-Mutant2.0, CUPSAT, PoPMuSiC, AUTO-MUTE2.0, SDM, DUET, mCSM, MAESTRO, and SDM2) and 3 sequential-based (I-Mutant2.0, MUpro, and iPTREESTAB) tools of stabilization's prediction. 4-letter PDB code or protein sequence in FASTA format is used as input, but the structural predictor achieves better performances than the sequential predictor. At the web server, http://ncblab.nchu.edu.tw/iStable2 can be found, the iStable 2.0.

### 2.6. Identification of Conserved Residues and Sequence Motifs

Sequence of human-CCBE1 protein UniProt showed markable comparison up to maximum of 100 sequences, and it was blasted against the UniProtKB/SwissProt database in NCBI (http://blast.ncbi.nlm.nih.gov/Blast.cgi). To perform, another computational analysis of the sequence was used, Clustal Omega. It showed more than 50% identity and E-value under 1, 00E-20 [39]. The amino acids identified were colored by scheme of Clustal color, and the alignment position conservation index was provided by Jalview [40].

### 2.7. Prediction of Amino Acid Conservation by ConSurf (ConSurf.tau.ac.il)

Bayesian empirical inference is used to calculate evolutionary conversation sequence of amino acid within a sequence of protein. This inference is giving us conservation scores along with schemes of color. Variable amino acid gets score 1, while the most conserved amino acid gets score 9. To ConSurf analysis was submitted the FASTA sequence of CCBE1 protein [41].

### 2.8. Project HOPE

Analysis of structural effects of the intended mutation is performed by the website Project HOPE. In cooperation with UniProt and DAS servers of prediction, the HOPE Project shows the mutated protein in an observable 3D structure. Project HOPE is the protein sequence used as the input source, and then the wild-type amino acid comparison of the structure is performed [42].

### 2.9. Secondary Structure Prediction by NetSurfP

In a fully folded protein, to identify the interaction interfaces or active sites is necessary knowledge of amino acid surface and accessibility of solvent. When the amino acid substitutions in such sites are noticed, then the affinity of binding is disturbed [43]. Binding affinity is also disturbed by catalytic activity when an enzyme is a protein. Surface and solvent accessibility, structural disorder, backbone dihedral angles, and secondary structure, for amino acid residues, can be effectively estimated by NetSurf-2.0. Protein sequences in FASTA format are utilized as input. They recruit deep neural nets that were trained on solved protein structures [43]. The availability of NetSurfP-2.0 is on the website http://www.cbs.dtu.dk/services/NetSurfP/.

### 2.10. Predicting 3D Protein Structure

The 3D homology modelling tool that can predict 3D models of proteins is called Phyre2 (http://www.sbg.bio.ic.ac.uk/∼phyre 2/html/page.cgi?xml:id = index) [44]. There were generated 3D models of wild-type CCBE1 with its 23 mutants associated with most deleterious nsSNPs. TM-align (https://zhanglab.ccmb.med.umich.edu/TM-align/) was used for comparison of the wild-type CCBE1 and selected mutants. There were predicted TM-score (template modelling score), RMSD (root-mean-square deviation) and structural superposition. The range of TM-scores is provided from 0 to 1, where 1 is identified as a higher structural similarity. The greater will be the variation between mutant and wild-type structures, the higher will be the RMSD values [45, 46]. To I-TASSER for further study of 3D protein structure study (https://zhanglab.ccmb.med.umich.edu/I-TASSE%20R/), were submitted 3 mutants with higher RMSD along with the wild-type CCBE1 [47, 48, 49]. Chimera v1.11 was used to investigate molecular characteristics and to visualize the resulting protein structure interactively [50].

### 2.11. PTM Site Prediction

Post‐translation modification (PTM) in protein is used to predict the function of the protein. GPS‐MSP v3.0 (http://msp.biocuckoo.org/online.php) was used to predicate methylation sites in CCBE1 protein [51]. At residual positions of serine, tyrosine, and threonine at CCBE1 sequence of protein, the prediction of phosphorylation sites is made by using GPS 3.0 (http://gps.biocuckoo.org/online.php) [52] and NetPhos 3.1 (http://www.cbs.dtu.dk/services/NetPhos/). By employing NetPhos 3.1 for neural network ensembles, a threshold of 0.5 was created, which predicted more specific findings than GPS 3.0 [53]. There was a prediction that residues having a higher score than threshold should be phosphorylated. To the prediction of ubiquitylation sites in CCBE1 protein were used BDM‐PUB (http://bdmpub.biocuckoo.org/prediction.php) and UbPred (http://www.ubpred.org/). UbPred had chosen a balanced cutoff [37] for lysine residues that were predicted ubiquitinated to have scored at or above the 0.62 thresholds [54]. NetOglyc4.0 (http://www.cbs.dtu.dk/services/NetOG%20lyc/) predicted glycosylation, which is another very important post-transcriptional event [55]. The website of NetOglyc4.0 is analyzing protein sequence with amino acid substitution and also a wild-type protein sequence. Mutation is functionally significant when there is difference between the functional pattern in mutant type and wild type. There is the prediction that glycosylation sites with higher score than threshold 0.5 will be glycosylated.

### 2.12. Ligand-Binding Site Prediction by FTSite Server

(http://FTSite.buedu/) Server FTSite has predicted the ligand-binding site in the 3D protein structure. Prediction of this site is based on energy, and the binding site over 94% of the apoproteins is identified. To the prediction of the hotspot, ligand-binding used PDB data as input.

### 2.13. Statistical Analysis

Computational in silico tool predication was subjected to correlation analysis using SPSS v23 and MS excel. The various computational tool prediction significance differences were compared using Student's *t*-test. A *p*value *<*0.01 was considered significant.

## 3. Results

### 3.1. Exploring the Desired Gene Using dbSNPs/NCBI

**Figure 1:**
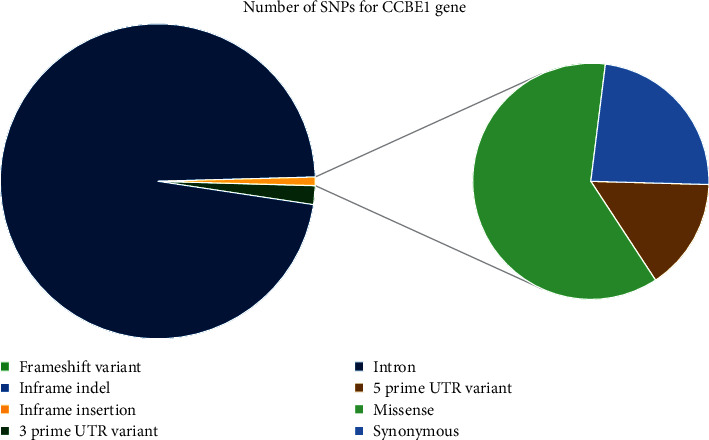
Distribution of all SNPs in CCBE1 gene.

CCBE1 gene SNP data were searched in the NCBI database (http://www.ncbi.nlm.nih.gov/). It contains a total of 73845 SNPs, which were present in *Homo sapiens*, 407 were found in nonsynonymous regions (missense), and 156 were in synonymous as shown in Figure 1.

### 3.2. GeneMANIA

**Figure 2:**
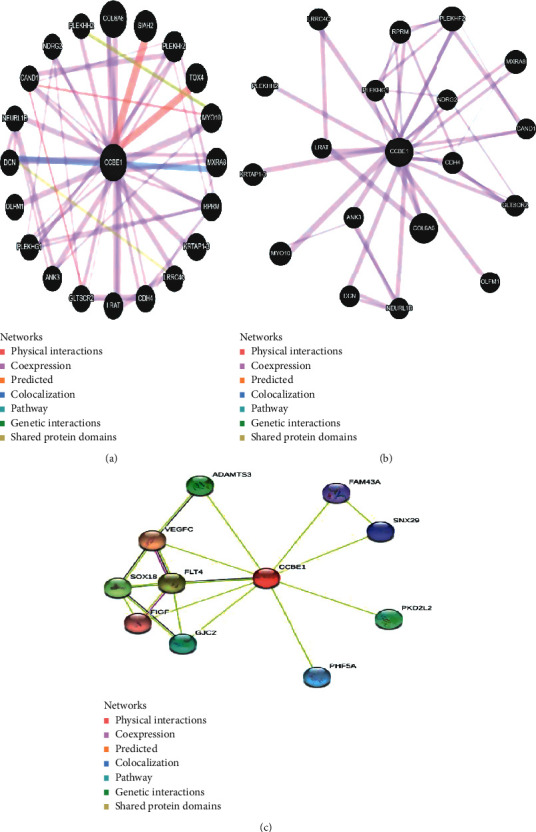
(a) Gene-gene interaction of CCBE1 with other genes proposed by GeneMANIA. (b) Coexpression in GeneMANIA. (c) Gene-gene interaction of CCBE1 with other genes proposed by STRING.

The CCBE1 gene provides instructions for making a protein that is found in the extracellular matrix of protein lattice and other molecules. The CCBE1 protein is involved in the formation of the lymphatic system. Specifically, the *CCBE1* protein helps guide immature cells called lymphangioblast maturation (differentiation) and movement (migration) that will eventually form the lining (epithelium) of lymphatic vessels. Our findings revealed that CCBE1 is coexpressed with 17 genes (COL6A6, MXRA8, PLEKHF2, RPRM, CDH4, PLEKHG1, CAND1, MY010, LRRC4C, LRAT, ANK3, OLFM1, DCN, NEURL1B, PLEKHH2, GLTSCR2, and NDRG2) and shared domain with only 2 genes (PLEKHH2 and DCN), physical interaction with two genes (SIAH2 and TOX4), and colocalization with 2 genes (MYRA8 and DCN). Predictions resulted from STRING showed combined score for each of the genes and showed interaction of the gene with FLT4, VEGFC, ADAMTS3, GJC2, FLGF, FAM43A, SNX29, PKD2L2, and PHF5A. Gene interactions predicted by GeneMANIA (Figures 2(a) and 2(b) and Table 1) and STRING (Figure 2(c)) are given in Figure 2, respectively.

### 3.3. Prediction of Deleterious nsSNP by SIFT and PolyPhen in CCBE1

A total of 407 nsSNPs (missense) were screened to find their effect on protein structure and function. The first step was to predict the nsSNP carried out the amino acid substitution. SIFT predicts the effect of nsSNP on protein structure and tells whether the induced amino acid is tolerable at that position or not. Out of a total of 407 nsSNPs, 23 were found to be deleterious with a tolerance index score of 0.00 on the SIFT network, as well as on prediction matching of highly pathogenic nsSNPs with a PSIC score of >0.5 on the PolyPhen server. There 11 nsSNPs contained the information of minor allele frequency (MAF). Except for T153N, G107D, P249S, S19N, C75S, C102S, G327 R, C174R, D397Y, R125W, P87W, and G330E, other MAFs of nsSNPs might be lower than 1% (Table 2).

#### 3.3.1. Confirmation of Delirious nsSNP by Different Tools in CCBE1

Fifteen in silico algorithms were used to confirm 23 deleterious/damaging nsSNPs predicated by SIFT and PolyPhen. These tools were used for confirmation analysis PROVEAN, FATHMM, LRT, M-CAP, VEST3, CAAD, MetaLR, DANN, Mutation Assessor, Mutation Taster, FATHMM-MKL, SNP-GO, PhD-SNP, PANTHER, and SNAP2. Any of the seventeen prediction tools was used independently or in combination with a tool that showed the effects of several prediction tools. Each method has a different number of deleterious SNPs. SIFT classified 36 and PolyPhen 23 nsSNPs as damaging or deleterious, but PolyPhen did not demonstrate any of the damaging 13 nsSNPs that SIFT classified as deleterious. With a cutoff of >0.5, SNP-GO revealed the fewest 4 SNPs (17.23%) in total of 23 SIFT- and PolyPhen-predicated nsSNPs in the CCBE1 gene as damaging or deleterious, and 19 as neutral. Using SNAP2 tool, 18 (78.26%) (09 effective nsSNPs : SNAP2 score 0 to 50; 09 highly effective: SNAP2 score 50 to 100) and 05 were neutral (SNAP2 score −100). The deleterious and damaging effects of 21 (91.23%) nsSNPs in which 18 nsSNPs probably damaging, 3 nsSNPs as possibly damaging, and 2 (8.6%) probably benign (time > 450my “possibly damaging,” 450my > time > 200my, “probably benign,” and time < 200my on CCBE1 protein), were predicted using the PANTHER (Figure 1 S4). Furthermore, the analysis was carried out using the PROVEAN, which predicts the impact of SNP on the biological function of a protein. A total of 11 (47.82%) nsSNPs of CCBE1 gene were predicted to be highly deleterious using PROVEAN having cutoff >−2.667 (Figure 1 S4), and 12 nsSNPs were neutral. Mutation Assessor predicates 3 nsSNPs high, 9 medium, 8 low, and 2 as neutral with a threshold of >0.65 (−5.545 to 5.975 (higher score‐>more damaging). FATHMM-MKL (<0.5), CADD (>15), and M-Cap (>0.025) with respective scores show all 23 (100%) nsSNPs as deleterious/damaging. DANN predicated 19 deleterious and 4 as tolerated with cutoff (>0.5). Mutation Taster with a threshold of (<0.5) predicated 21 (91.30%) as deleterious and 2 as polymorphic while VEST3 predicated 15 (65.21%) deleterious and 8 tolerated with a cutoff (<0.5). FATHMM with a score of (>0.453) predicated 17 (73.91%) nsSNPs deleterious and 5 as tolerated, while LRT predicated 19 (82.60%), with score >0.001, nsSNPs deleterious and 4 as neutral. PhD-SNP showed 13 (56.56%) deleterious SNPs and 10 neutral. FATHMM-MKL Furthermore, on the PolyPhen server, prediction matching of highly pathogenic nsSNPs was carried out with PSIC score (>0.5). A group of 4 nsSNPs, rs149531418 (G330E), rs121908251 (C102S), rs121908254 (C174R), and rs372499913 (G107D), were cumulatively considered as highly deleterious as these 4 nsSNPs were supported 100% by all of the state-of-the-art tools while only Mutation Assessor disagrees with the result of G107D by other tools. Even though the SNAP2 agreed with G330E, C102S, and C174R as effect, the score is <50 (Table 1S4). During the prediction matching analysis, the nsSNPs, rs149531418 (G330E), rs121908251 (C102S), rs121908254 (C174R), and rs372499913 (G107D), were agreed by the state-of-the-art tools, PolyPhen (>0.5), PANTHER (>450), SNPs&GO (>0.5), SIFT (=0), Mutation Taster (<0.5), CADD (>15), MetaLR (>0.5), M-CAP (>0.025), PANTHER (probably damaging time > 450my possibly damaging” (450my > time > 200my, “probably benign” (time < 200my)), VEST3 (>0.5), LRT (>0.001), PROVEAN (>−2.667), FATHMM-MKL (<0.5), PhD-SNP (>0.5), SNP-GO (>0.5), SNAP2 (−100 (fully neutral) and +100 (strong effect)), DANN (>0.5), Mutation Assessor (>0.65) (−5.545 to 5.975 (higher score‐>more damaging)), FATHMM (>0.453), and highly deleterious nsSNPs on CCBE1 gene. Analysis of 407 nsSNPs of CCBE1 gene for the prediction of pathogenic nsSNPs was almost similar (87%) for the SIFT and PolyPhen while disagreement was 36%. We selected for further study 23 nsSNPs which were predicated deleterious/damaging by both SIFT and PolyPhen. More than 100% of overlapped similarity was observed between the SIFT, M-CAP, CADD, PolyPhen, and FATHMM-MKL, on pathogenic nsSNPs. Similarity between SNP-GO and PhD-SNP is 13%, and disagreement is 73% while between SIFT and SNP-GO dissimilarity was 82%. Almost more than 50% of the predictions of pathogenic nsSNPs were found to be disagreed between SIFT, and PROVEAN, SNAP2, PANTHER, MetaLR, Mutation Assessor, FATHMM, VEST3, and MutPred. Moreover, similarities in between these tools (SNAP2, MetaLR, Mutation Taster, DANN, FATHMM, and LRT) for predication were more than 70%. Almost 60% agreement for pathogenic nsSNPs was present in predication tools (MutPred, VEST3, PhD-SNP, and Mutation Assessor). The results of all the predication algorithms were found statistically significant and were highly correlated. Student's *t*-test between the tools was significant at *p* value *<*0.001. The results are shown in Table 3 as well as the cumulative score and total significance of all the tools in the study are shown in Figure 1S4.

### 3.4. Conservation Analysis

**Figure 3:**
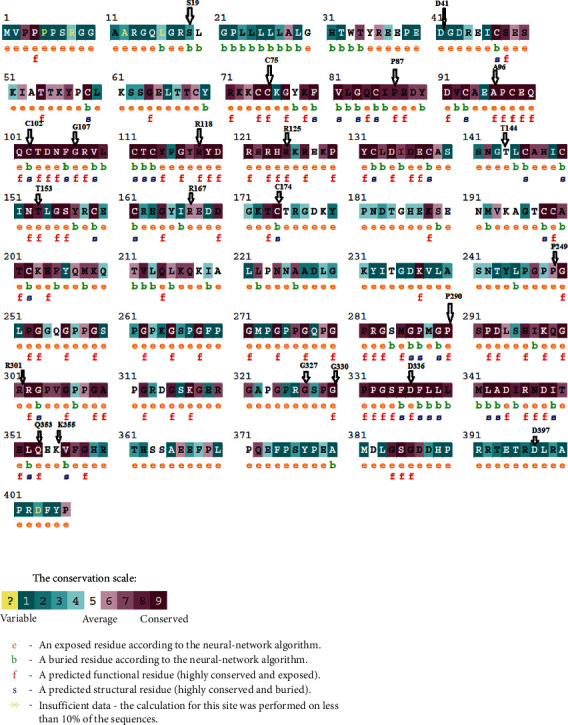
Evolutionary conservation of amino acids in the ADA gene determined by the ConSurf server. Value 1 indicates a high variability region. The value increases as the region becomes more conserved, up to value 9.

We analyzed the degree of conservations of CCBE1 residues by using the ConSurf web server. The results of the ConSurf analysis indicated that 23 deleterious missense SNPs are located in highly conserved regions (7-8-9). Among these 23 missenses variants, 13 were located in the highly conserved positions: 11 (C75S, P87S, P290L, A96G, G107D, R118L, G330E, D336N, R125W, Q353R, and T153N) were predicted as functional and exposed residues and the other 2 (C102S and C174R) were predicted as buried and structural residues. The S19N was predicted as conserved and buried residue, and the other 8 (T144M, R167W, P249S, R301W, G327R, K355T, D397Y, and D41E) were exposed residues. The results are shown in Figure 3.

### 3.5. Project Hope

All of the 23 nonsynonymous SNPs that were predicted to be deleterious and damaging by both SIFT and PolyPhen software were submitted to Project HOPE software. The findings revealed that rs149531418 resulted in the substitution of glycine (wild type) into glutamic acid (mutant) at position 330. The mutant residue is bigger than the wild-type residue. The wild-type residue charge was neutral, and the mutant residue charge was negative. The wild-type residue is more hydrophobic than the mutant residue as well as the mutation is located within a domain, annotated in UniProt as collagen-like 2, and the mutation introduces an amino acid with different properties, which can disturb this domain and abolish its function. Neither our mutant residue nor another residue type with similar properties was observed at this position in other homologous sequences. Based on conservation scores, this mutation is probably damaging to the protein. The mutant residue is located near a highly conserved position. The rs121908251 resulted in the substitution of cysteine (wild type) into serine (mutant type) at position 102. The wild-type residue is more hydrophobic than the mutant residue. The variant is annotated with severity: disease, and the mutation is located in a region with known splice variants, described as C- > S (in HKLLS1; dbSNP: rs121908251). The mutant and wild-type residues are not very similar. Based on this conservation information, this mutation is probably damaging to the protein. This mutant residue is located near a highly conserved position. The rs121908254 shows the substitution of cysteine (wild type) into arginine (mutant type) at position 174. The mutant residue is bigger than the wild-type residue. The wild-type residue charge was neutral, and the mutant residue charge was positive. The wild-type residue is more hydrophobic than the mutant residue. The mutation is located within a domain, annotated in UniProt as EGF-like, calcium-binding. The mutation introduces an amino acid with different properties, which can disturb this domain and abolish its function. The variant is annotated with severity: disease, and mutation is located in a region with known splice variants, described as C- > *R* (in HKLLS1; dbSNP: rs121908254). The mutant and wild-type residues are not very similar. Based on this conservation information, this mutation is probably damaging to the protein. The mutant residue is located near a highly conserved position. The rs372499913 indicates the substitution of glycine (wild type) into aspartic acid (mutant type) at position 107. The mutant residue is bigger than the wild-type residue. The wild-type residue charge was neutral, and the mutant residue charge was negative. The wild-type residue is more hydrophobic than the mutant residue. The mutant and wild-type residues are not very similar. Based on this conservation information, this mutation is probably damaging to the protein. Our mutant residue is located near a highly conserved position. SNP rs147208835 results in the substitution of arginine (wild type) into tryptophan (mutant type) at position 125. The mutant residue is bigger than the wild-type residue. The wild-type residue charge was positive, and the mutant residue charge was neutral. The mutant residue is more hydrophobic than the wild-type residue. The mutant residue was not among the other residue types observed at this position in other homologous proteins. However, residues that have some properties in common with your mutated residue were observed. This means that in some rare cases, your mutation might occur without damaging the protein. The mutant residue is located near a highly conserved position.

### 3.6. Association of SNPs with Highly Conserved Buried (Structural) and Exposed (Functional) Amino Acid Residues in CCBE1 Protein

**Figure 4:**
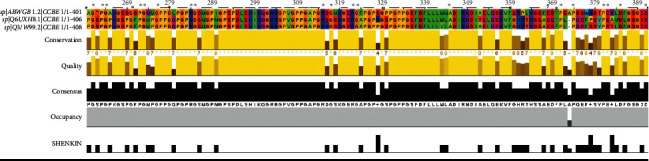
Amino acid alignment of human CCBE1 (UniProt ID: Q6UXH8) along with its homologues in phylogenetically close species in ABWGB and Q3MI99. Solid horizontal bars indicate conserved sequence motifs, and residues with asterisk (^∗^) mark indicate evolutionary conserved amino acids. The amino acid identities were colored according the Clustal color scheme, and the conservation index at each alignment position was provided by Jalview.

CCBE1 from a structural point of view expresses as a 406 amino acid long protein having 11 exons located at 18q21.32. CCBE1 sequence-based structural-functional analysis was performed using Clustal Omega-based multiple sequence alignment analysis. For this analysis, the CCBE1 protein sequence (UniProt ID: Q6UXH8) was retrieved from the UniProt Knowledgebase. The CCBE1 protein sequence was blasted against the UniProtKB/SwissProt entries and aligned using Clustal Omega with default settings. The results generated by the Clustal Omega tool consist of CCBE1 protein sequence aligned with other phylogenetically close sequences from other organisms. The results contain a colorimetric conservation score in the range of 1–10. Multiple sequence alignment using Clustal Omega revealed that the human CCBE1 protein sequence contains a number of conserved residues and motifs. The highly conserved amino acid residues in human CCBE1 protein were G262, P264, G265, G270, P272, G273, G276, R284, G285, R315, G317, R322, G323, G329, A345, E368, F370, P371, P374, P381, E382, D385, and D391. There are twenty-four different conserved residues Figure 4.

### 3.7. Prediction of Pathogenic Amino Acid Substitutions by MutPred2

MutPred2 considers several molecular characteristics of amino acid residues to predict whether an amino acid substitution is disease-related or neutral in humans. The score it provides is the probability predicted for an amino acid substitution should affect the function of the respective protein or not. The threshold score for pathogenicity prediction is 0.5, and a MutPred2 score ≥0.8 can be considered as a highly confident one. All substitutions have prediction scores ≤0.5.  Table 4 provides MutPred2 outcomes.

### 3.8. Prediction of Stability of the Mutated Protein due to SNPs by iStable 2.0

Web tool iStable 2.0 was used to analysis for protein stability prediction. This web tool consists of 11 sequence- and structure-based prediction tools, and a machine learning approach is used for all outputs. Mutations were run from sequence analysis due to the unavailability of experimental structure. The results showed that G330E, C174R, G327R, P290L, D41E, A96G, T114M, D397Y, S19N, and Q359RT have increased stability while P249S, R167W, R301W, C75S, P87S, R118L, T153N, D336N, R125W, K355T, G107D, and C102S showed decreased stability.  Table 5 provides iStable 2.0 predictions.

### 3.9. Surface and Solvent Accessibility of Residues and CCBE1 Secondary Structure by NetSurfP-2.0

**Figure 5:**
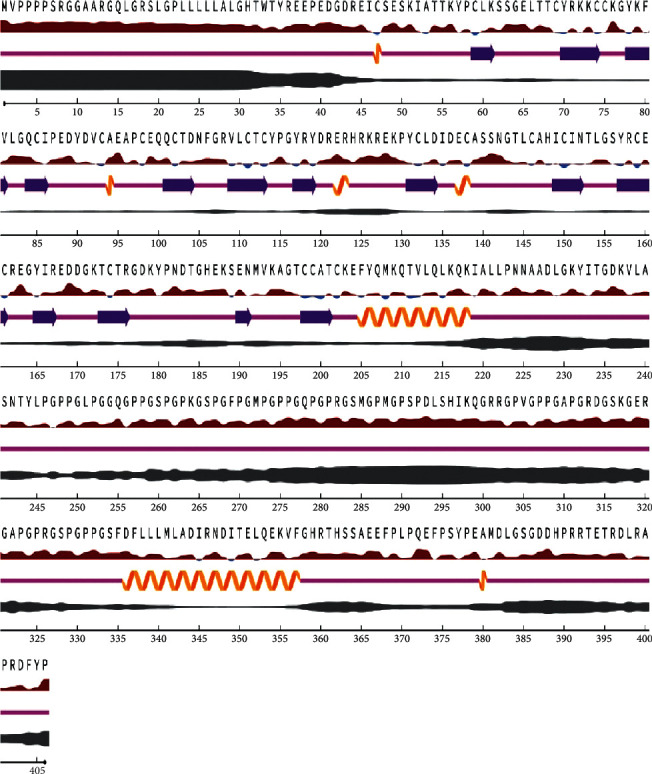
Secondary structure predication by NetSurfP-2.0.

Surface accessibility (exposed or buried) of amino acids in a given protein was predicated by NetSurfP-2.0, which provides a relative and absolute accessible surface area of each residue. It also predicts the protein secondary structure. Relative surface accessibility: red upward elevation is exposed to residue, and sky blue downward elevation is buried residue; the threshold is at 25%. Secondary structure is as follows: orange spiral = helix, indigo arrow = strand, and pink straight line = coil. Disorder is represented as black swollen line; thickness of line equals the probability of disordered residue.  Figure 5 shows NetSurfP-2.0 outcomes.

### 3.10. 3D Modelling of CCBE1 and Its Mutants

**Figure 6:**
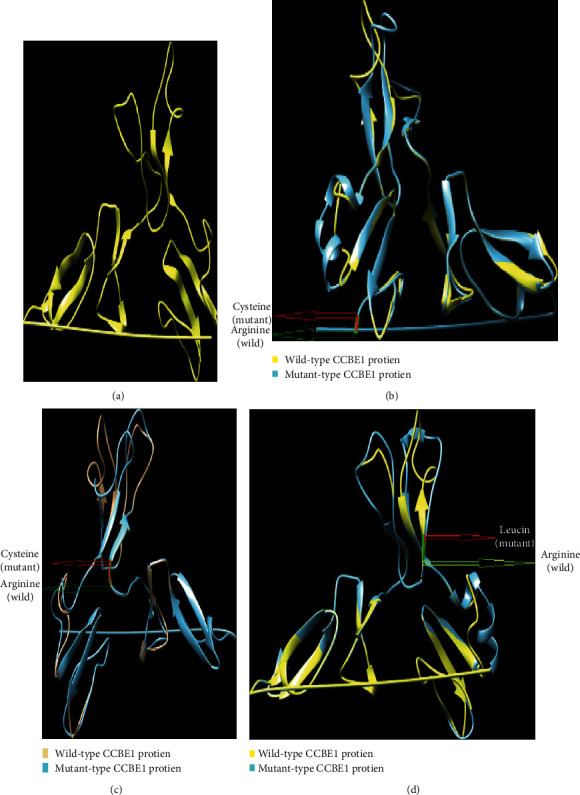
(a) Wild-type CCBE1 protein structure. (b) Superimposed structure of CCBE1 and its C174R mutant. (c) Superimposed structure of CCBE1 and its A96G mutant. (d) Superimposed structure of CCBE1 and its R118L mutant.

Phyre2 was used for 3D structure generation of wild‐type CCBE1 protein and 22 mutants. For generating mutant protein 3D structure, nsSNP substitutions were made individually in CCBE1 protein sequence and then submitted to Phyre2, which predicted their 3D structures. Phyre2 used c5to3B as a template for 3D model prediction because it was the highest similar template according to the Phyre2 server. TM‐scores and RMSD values were calculated for each of the mutant models. The TM‐score shows us the topological similarity while RMSD values show the average distance between *α*‐carbon backbones of wild and mutant models. Higher RMSD values predict greater mutant structure deviation from wild type. The model for the mutant R118L (rs115982879) showed the greatest deviation having 1.56B RMSD value followed by A96G (rs149792489), S19N (rs374941368), and C174R (rs121908254) with 1.50B, 1.44B, and 1.46B RMSD values, respectively. R125W, C75S, and T153N showed 0.89B, 0.90B, and 0.85B RMSD values, thus showing no variation in structure from wild type. Other nsSNPs showed slight variation which included G327R (1.36B RMSD), P290L (1.3.6B RMSD), Q353T (1.3.2B RMSD), P290L (1.25B RMSD), D336N (1.25B RMSD), C102R (1.22B RMSD), R167W (1.16B RMSD), P87L (1.14B RMSD), G107D (1.13B RMSD), T144M (1.13B RMSD), G330R (1.12B RMSD), D41E (1.12B RMSD), D297Y (1.06B RMSD), R301W (1.02B RMSD), and K355T (1.01B RMSD). TM‐scores and RMSD values are given in Table 6. Four nsSNPs (R118L, A96G, S19N, and C174R) having the highest RMSD values were selected and submitted to I‐TASSER for remodeling. Protein structure generated by the I‐TASSER is the most reliable as it is the most advanced modelling tool. Each of these 3 mutants was studied and superimposed using Chimera 1.11 over the wild‐type CCBE1 protein, shown in Figures 6(a)–6(d).

### 3.11. Predicted PTMs (Post‐Translation Modifications)

GPS‐MSP 3.0 was used for this purpose which predicted no sites in CCBE1 to be methylated. GPS 3.0 and NetPhos 3.1 predicted CCBE1 phosphorylation sites which are given in Table S1. 62 residues (Ser: 23, Thr: 22, and Tyr: 17) were predicted by NetPhos 3.1 to have phosphorylation potential. On the other hand, 18 residues (Ser: 12, Thr: 06, and Tyr: 00) were predicted by GPS 3.0 to be capable of getting phosphorylated. BDM‐PUB and UbPred were used for ubiquitylation prediction. BDM-PUB predicted 11 lysine residues to get ubiquitinated, while UbPred predicted none of the lysine residues to get ubiquitinated. Among those predicted by BDM‐PUB, none was located at a highly conserved or deleterious nsSNP region. The results obtained are labeled in Table S1. NetOGlyc4.0 was used for the prediction of potential glycosylation sites. The output showed all the possible sites for glycosylation in which positions 19, 144, and 153 were predicted to be glycosylated with scores of 0.34, 0.43, and 0.17 in wild‐type CCBE1 protein. Interestingly, mutant S19N showed loss of glycosylation site at position 19 while T144M also showed loss of glycosylation sites at position 144. All the scores for the wild‐type and mutant proteins are given in Table S2.

### 3.12. Ligand-Binding Site Prediction by FTSite

**Figure 7:**
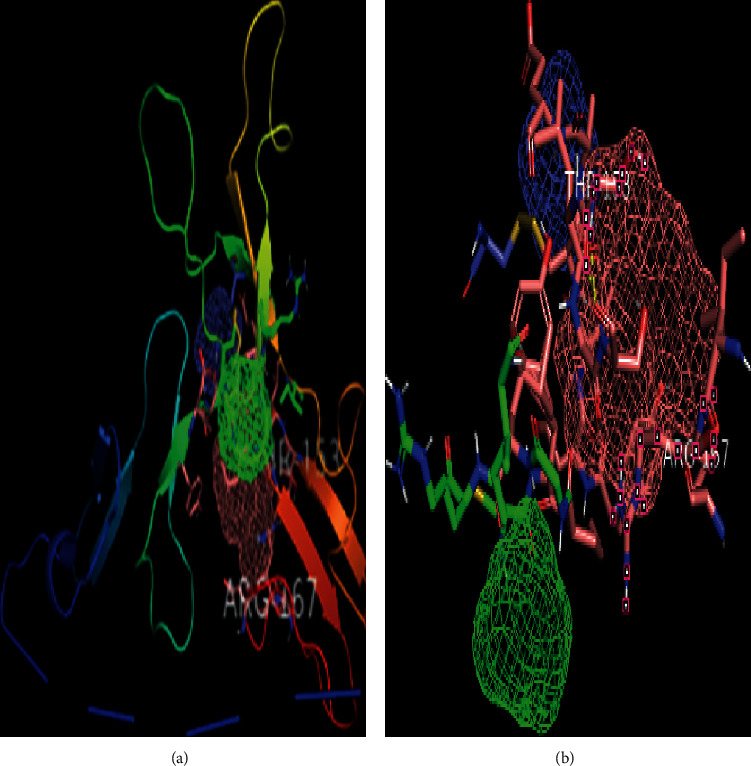
(a) Ligand-binding site prediction by FT site in whole predicated 3D protein model of CCBEI gene. (b) 3 meshes were predicted as ligand-binding site.

Sites for ligand-binding were predicted by FTSite algorithms and visualized and further analyzed using PyMOL. By this tool, 3 ligand-binding sites were identified in human CCBE1 protein (Figures 7(a) and 7(b)). Site 1 consisted of 14 residues; site 2 and site 3 consisted of 7 and 5 residues. Some of the substitutions in twenty-two substituted positions predicted by the SIFT server lie in the predicted ligand-binding sites (T153N and R167W) (Table S3).

## 4. Discussion

Several studies have linked the CCBE1 gene to single-nucleotide polymorphisms in the cases of lymph vessel dysplasia [13, 14]. Utilizing state-of-the-art in silico methods, the current research explored the impact of SNPs on the structural and interactive behaviors of the CCBE1 protein. The most pathogenic polymorphisms in different genes have been screened using these methods in a sequential order [42, 56]. The current study also used the sequential application of all these methods to classify deleterious variants in CCBE1 that may interact with the machinery's role in extracellular matrix remodeling and migration by silencing its function. We screened 73845 SNPs in the CCBE1 gene through multiple dbSNP databases for their effect on the gene's structure and interactions with a variety of protein molecules. Various in silico methods were used to screen the pathogenicity of 407 retrieved nonsynonymous SNPs. Our study found 23 nsSNPs that were predicted to be deleterious by SIFT and PolyPhen2 but instead verified through other tools (PROVEAN, FATHMM, LRT, M-CAP, VEST3, CAAD, MetaLR, Mutation Assessor, Mutation Taster, and FATHMM-MKL, SNP-GO, PhD-SNP, PANTHER, SNAP2, and MutPred). Four nsSNPs were classified as highly pathogenic which were rs149531418, rs121908251, rs121908254, and rs372499913. This is a lower number than which was previously estimated using the same methods in different genes [56, 57]. The two of the variant shown in our study (C102S, C174R) are already reported for Hennekam syndrome in a study [11], while the other two variants (G330E and G107D) are not reported until now for Hennekam syndrome. Highly pathogenic variants were selected on the basis of the impact of nsSNPs on sequence conservation, sequence attributes, and structural impute [58]. The chosen state-of-the-art tools covered the largest possible range of methods (AS: alignment score; NN: neural networks; HMM: hidden Markov models; SVM: support vector machine; BC: Bayesian classification) for predicting pathogenic nsSNPs [58]. Since essential amino acids that are involved in a wide range of biological methods and processes, particularly protein interactions, are highly modified and conserved, SNPs on conserved loci are more likely to cause damage than SNPs on nonconserved loci [59]. In total 23 nsSNPs, only 11 SNPs are located at evolutionary conserved, exposed, and functionally important residues which are C75S, P87S, P290L, A96G, G107D, R118L, G330E, D336N, R125W, Q353R, and T153N. There were 2 nsSNPs (C102S and C174R) located at conserved, buried, and structurally important residues. All the rest of the nsSNPs were found to be located in either only exposed or buried residues which were not predicted to have any structural or functional importance in CCBE1 protein. These 11 nsSNPs for CCBE1 have not yet reported with patients in Hennekam disorder, and in future, these can be considered pathogenic nsSNPs when reported in Hennekam patients. For prediction of protein stability, I‐STAB2 web server was used which predicted nsSNP rs149531418, rs121908254, rs147681552, rs192224843, rs147974432, rs141125426, rs374941368, and rs149792489 increased stability while C75S, P87S, R125W, K355T, D336N, T153N, P87S, R118L, R301W, P249S, and R167W decrease protein stability. These nsSNPs can be used as marker for diagnostic and revealing new therapeutic targets for Hennekam disorder. RAMPAGE values were used to verify all of the modeled structures. Protein structures with RAMPAGE values greater than 80% as core values are thought to be higher [60]. For the structure given in Figure 5(a) (CCEB1 wild‐type), RAMPAGE values were 75.5% favored residues, 19.1% allowed, 4.5% generally allowed, and disallowed 0.9%. Similarly, for mutants R118L (80.0% favored residues, 13.6% allowed, 4.5% generally allowed, and disallowed 1.8%), A96G (76.4% favored residues, 16.4% allowed, 5.5% generally allowed, and disallowed 1.8%), C174R (79.1% favored residues, 15.5% allowed, 2.7% generally allowed, and disallowed 0.9%), and S19N (78.2% favored residues, 16.4% allowed, 4.5% generally allowed, and disallowed 0.9%), all the structures were somehow validated. PTMs have been shown to be important in cell signaling and protein-protein interactions, as well as other significant events such as biological processes, control protein structures, and functions [61, 62]. In this analysis, we looked to see if the chosen nsSNPs modified the PTMs of the CCBE1 protein. A variety of bioinformatics methods were used to predict PTM sites in our understudied protein. Methylation is a critical PTM because lysine residues in some proteins are methylated, which influences their binding to DNA and changes gene expression. Another important mechanism for protein regulation acts as a molecular switch of protein to adapt it for functions such as protein structure conformational changes, protein activation and deactivation, and signal transduction pathways [63–66]. S19 is highly conserved, exposed, and functionally significant, according to the ConSurf conservation profile, indicating its significance. Phosphorylation potential is seen at position S19, which also contains one of the most damaging nsSNPs (rs137 6162684), which really is structurally important and highly conserved (ConSurf prediction), making it highly important. Ubiquitylation is a protein degradation mechanism that also helps in DNA damage repair [67]. It is crucial to the function and stability of proteins. It plays a structural role in protein-protein interactions. Phosphorylation is the only PTM that can have a major impact on CCBE1 protein structure and function, as shown by these PTM predictions, with residuals S19 and T153 being the most significant phosphorylation sites. STRING and GeneMANIA predictions show that ADAMTS3 is the most interactive gene with CCBE1, supported by VEGFC and FLT4. CCBE1 ADAMTS3, VEGFC, FLTR4, and GJC2 are thought to be related with either Hennekam disorder or its related symptoms in many diseases, including rheumatoid arthritis [8, 13, 68, 69]. As a result of their interaction patterns and coexpression profiles, it can be inferred that some of the most harmful nsSNPs in the CCBE1 gene will influence and possibly disrupt the normal functioning of other interacting genes. This demonstrates the significance of these interacting and coexpressing genes, which may be significant during the Hennekam syndrome or other primary immunodeficiency disorders. FTSite was used to look into the impact of substitutions on protein function. The FTSite server predicted three ligand-binding sites, each with 14, 7, and 9 residues. We discovered that R167W and T153N substitutions are involved in the ligand-binding site and form the catalytic coordination sphere, which can affect the CCBE1 protein's binding affinity. Since our research was thorough, it contains all of the necessary data and analysis for identifying the most harmful nsSNPs. Any research, including ours, has some limitations. The focus of our research is on mathematical and computational algorithms used in programming tools and web servers. As a consequence, experimental research is needed to confirm these findings. Our findings shed light on the CCBE1 gene's nsSNPs, protein 3D structure, PTM potential sites, and gene-gene interaction, and all of which may help researchers better understand the gene's role in autoimmunity and related diseases in the future.

## 5. Conclusion

The impact of nsSNPs on the functional and structural deviations in the CCBE1 protein was predicted using a variety of various state-of-the-art tools. On the CCBE1 protein, structural homology-based methods and sequence homology-based techniques have identified four nsSNPs as potentially damaging: rs149531418 (G330E), rs121908251 (C102S), rs121908254 (C174R), and rs372499913 (G107D). The pathogenicity of nsSNPs can be predicted in a stepwise and accurate manner (SIFT > PolyPhen > CADD > FATHMM-MKK > M-CAP > PANTHER > Mutation Taster > LRT > DANN > MetaLR > SNAP2 > VEST3> MutPred > PhD-SNP > Mutation Assessor > PROVEAN > SNP-GO > Cumulative), prediction matching among the tools. As a consequence, the findings of these tools for other studies may be considered more reliable. The importance of rs374941368 and rs200149541 in the prediction of post-transcriptional modifications was highlighted because it affects a possible phosphorylation location. In the future, the 4 reported extremely deleterious, protein stability decreasing, and nsSNPs in highly conserved positions could be used as Hennekam syndrome marker nsSNPs. Even though we performed a thorough in silico study, further research is needed to fully understand the impact of these nsSNPs on protein structure and function.

## Figures and Tables

**Table 1:** Genes coexpressed and sharing a domain with CCBE1.

Gene symbol	Description	Coexpression	Shared domain
*COL6A6*	Collagen type VI alpha 6	Yes	No
*MXRA8*	Matrix remodeling associated 8	Yes	No
*PLEKHF2*	Pleckstrin homology and FYVE domain containing 2	Yes	No
*RPRM*	Reprimo, TP53 dependent G2 arrest mediator candidate	Yes	No
*CDH4*	Cadherin 4	No	No
*PLEKHG1*	Pleckstrin homology and RhoGEF domain containing G1	Yes	No
*CAND1*	Cullin associated and neddylation dissociated 1	Yes	No
*MYO10*	Myosin X	Yes	No
*LRRC4C*	Leucine rich repeat containing 4C	Yes	No
*LRAT*	Lecithin retinol acyltransferase	Yes	No
*ANK3*	Ankyrin 3, node of Ranvier	Yes	No
*OLFM1*	Olfactomedin 1	Yes	No
*DCN*	Decorin	Yes	Yes
*NEURL1B*	Neuralized E3 ubiquitin protein ligase 1B	Yes	No
*PLEKHH2*	Pleckstrin homology, MyTH4, and FERM domain containing H2	Yes	Yes
*GLTSCR2*	Glioma tumor suppressor candidate region gene 2	Yes	No
*NDRG2*	NDRG family member 2	Yes	No

**Table 2:** nsSNPs predicted as deleterious by SIFT and PolyPhen2.

ID of nsSNPs	AA position	SIFT	Score	PolyPhen2	Score	MAF
rs199902030	D336N	Deleterious	0.003	Probably damaging	1	<0.001 (T)
rs200149541	T153N	Deleterious	0.001	Probably damaging	1	
rs372499913	G107D	Deleterious	0	Probably damaging	1	
rs267605221	P249S	Deleterious	0.007	Probably damaging	1	
rs374941368	S19N	Deleterious	0.004	Probably damaging	0.981	
rs375717418	R301W	Deleterious	0.004	Probably damaging	1	<0.001 (T)
rs80008675	D41E	Deleterious low	0.016	Probably damaging	0.982	0.017 (T)
rs116596858	P181S	Deleterious low	0.007	Probably damaging	0.906	<0.001 (A)
rs116675104	R167W	Deleterious low	0.017	Probably damaging	0.990	0.003 (A)
rs121908250	C75S	Deleterious low	0.002	Probably damaging	0.981	
rs121908251	C102S	Deleterious low	0	Probably damaging	0.999	
rs121908252	G327R	Deleterious	0	Probably damaging	1	
rs121908254	C174R	Deleterious	0.001	Probably damaging	0.984	
rs147974432	T144M	Deleterious low	0.002	Probably damaging	1	<0.001 (A)
rs192224843	Q353R	Deleterious	0.011	Probably damaging	0.993	<0.001 (C)
rs115982879	R118L	Deleterious low	0.001	Probably damaging	0.910	<0.001 (T)
rs139059968	K355T	Deleterious	0.002	Probably damaging	0.883	<0.001 (G)
rs141125426	D397Y	Deleterious low	0.002	Probably damaging	0.828	
rs147208835	R125W	Deleterious low	0	Probably damaging	0.995	
rs147681552	P290L	Deleterious	0.005	Probably damaging	1	<0.001 (A)
rs148498685	P87S	Deleterious low	0.002	Probably damaging	1	
rs149531418	G330E	Deleterious	0	Probably damaging	0.999	
rs149792489	A96G	Deleterious low	0.004	Probably damaging	1	<0.001 (C)

*Threshold*. SIFT: <0.05; PolyPhen2: >0.8 (PSIC > 0.5) or Benign (PSIC < 0.5).

**Table 3:** Confirmation of the deleterious nsSNPs by other prediction software.

AAS	LRT	Mutation Taster	Mutation Assessor	PROVEAN	FATHMM	VEST3	MetaLR	M-CAP	CADD	DANN	FATHMM-MKK	PhD-SNP	PANTHER	SNP-GO	SNAP2
G330E	D	D	H	D	D	D	D	D	D	D	D	D	D	D	E
C102S	D	D	M	D	D	D	D	D	D	D	D	D	D	D	E
C174R	D	D	H	D	D	D	D	D	D	D	D	D	D	D	E
G107D	D	D	L	D	D	D	D	D	D	D	D	D	D	D	E
R125W	D	D	L	D	D	T	D	D	D	D	D	D	D	N	E
G327R	D	D	H	D	D	D	D	D	D	D	D	N	D	N	E
P290L	D	D	M	D	T	D	D	D	D	D	D	N	D	N	E
K355T	D	D	M	N	D	D	D	D	D	D	D	D	D	N	E
Q353R	D	D	M	N	D	D	D	D	D	D	D	D	D	N	E
D336N	D	D	M	N	D	T	D	D	D	D	D	D	D	N	E
T153N	D	D	M	N	D	T	D	D	D	D	D	D	D	N	E
C75S	D	D	L	D	D	D	D	D	D	T	D	N	D	N	E
P87S	D	D	L	N	D	D	D	D	D	D	D	D	D	N	E
T144M	D	D	L	N	D	D	D	D	D	D	D	N	D	N	E
R118L	D	D	L	D	D	D	T	D	D	D	D	D	D	N	E
D397Y	N	D	M	D	D	T	D	D	D	T	D	D	D	N	E
R301W	D	D	M	D	T	D	T	D	D	D	D	N	D	N	E
P249S	D	D	M	N	T	T	D	D	D	D	D	N	D	N	N
D41E	D	P	L	N	D	T	T	D	D	T	D	D	D	N	N
S19N	N	P	L	N	D	T	D	D	D	D	D	N	D	N	N
R167W	N	D	L	N	D	T	D	D	D	D	D	N	N	N	E
A96G	D	D	L	N	T	D	T	D	D	D	D	N	D	N	N
P181S	N	D	L	N	T	D	T	D	D	T	D	N	N	N	N

D: deleterious; T: tolerated; U: unknown; L: low; N: neutral; M: medium; P: polymorphism; E: effect. Thresholds for all these predication tools are given in the S4 fill.

**Table 4:** Predication of disease-related AA substitution and phenotypes by Mutpred2.

SNPs	Actionable/confident hypothesis	Probability	*p* value
C174R	Gain of intrinsic disorder	0.39	0.009
	Loss of disulfide linkage at C174	0.21	0.020

D336N	Altered disorder interface	0.29	0.02
	Loss of loop	0.26	0.05
	Loss of proteolytic cleavage at D336	0.11	0.05
	Altered coiled coil	0.11	0.04

G107D	Altered transmembrane protein	0.29	0.0003
	Loss of loop	0.27	0.02
	Loss of disulfide linkage at C102	0.26	0.004
	Gain of proteolytic cleavage at R108	0.15	0.01

C102S	Loss of disulfide linkage at C102	0.55	0.0003
	Loss of helix	0.28	0.03
	Loss of pyrrolidone carboxylic acid at Q100	0.19	0.002
	Altered metal binding	0.35	0.008
	Altered transmembrane protein	0.32	0.00007

G330E	Loss of B factor	0.27	0.02
	Gain of loop		0.04

G327R	Loss of B factor	0.27	0.02

P290L	Altered disordered interface	0.36	0.008
	Loss of B factor	0.29	0.01

Q353R	Altered disordered interface	0.29	0.03
	Altered coiled coil	012	0.04

T153N	Loss of strand	0.26	0.04
	Gain of disulfide linkage at C150	0.23	0.01

C75S	Altered metal binding	0.40	0.006
	Loss of disulfide linkage at C75	0.30	0.001
	Loss of helix	0.27	0.05

P87S	Gain of helix	0.28	0.02
	Gain of disulfide linkage at C85	0.20	0.02
	Altered metal binding	0.25	0.03
	Loss of sulfation at Y90	0.09	0.003

R118L	Altered disordered interface	0.27	0.40
	Loss of disulfide linkage at C113	0.19	0.02
	Gain of photolytic cleavage at D120	0.16	0.009
	Loss of sulfation at Y114	0.02	0.02

P181S	Gain of phosphorylation at Y180	0.37	0.007
	Loss of acetylation at K179	0.21	0.03
	Gain of N-linked glycosylation at N182	0.03	0.03

A96G	Altered transmembrane protein	0.30	0.0001
	Loss of helix	0.29	0.02
	Gain of disulfide linkage at C98	0.25	0.006
	Gain of pyrrolidone carboxylic at Q100	0.20	0.002

P249S	Gain of loop	0.31	0.004
	Loss of B factor	0.29	0.02
	Gain of phosphorylation at Y244	0.24	0.04
	Gain of o-linked glycosylation at P249	0.12	0.04

**Table 5:** iStable 2.0 result for prediction of CCBE1 protein stability due to the selected nsSNPs in CCBE1.

AAS	Confidence score	Stability
G330E	−0.002680719	Increase
C174R	0.021838337	Increase
C102S	−1.2213084	Decrease
G107D	−0.86388123	Decrease
R125W	−0.85255766	Decrease
G327R	0.0042461157	Increase
P290L	0.2298831	Increase
K355T	−0.052274585	Decrease
Q353R	0.8725257	Increase
D336N	−1.2082165	Decrease
T153N	−0.546193	Decrease
C75S	−1.0542232	Decrease
P87S	−1.9976869	Decrease
T144M	0.23297998	Increase
R118L	−0.5704589	Decrease
D397Y	0.071232796	Increase
R301W	−0.3441298	Decrease
P249S	−1.1325055	Decrease
D41E	0.4703572	Increase
S19N	0.77003396	Increase
R167W	−0.4350294	Decrease
A96G	−0.041893244	Increase

**Table 6:** TM‐score and RMSD values of 23 selected damaging nsSNPs in CCBE1.

SNP-ID	Residual change	TM-score	RMSD values	SNP-ID	Residual change	TM-score	RMSD values
rs199902030	D336N	0.92174	1.25	rs121908252	G327R	0.91250	1.36
rs200149541	T153N	0.95561	0.84	rs121908254	C174R	0.92822	1.46
rs372499913	G107D	0.95388	1.13	rs147974432	T144M	0.93348	1.13
rs267605221	P249S	0.91250	1.36	rs192224843	Q353R	0.92957	1.32
rs374941368	S19N	0.92526	1.44	rs115982879	R118L	0.92844	1.57
rs375717418	R301W	0.93696	1.02	rs139059968	K355T	0.93921	1.01
rs80008675	D41E	0.95466	1.12	rs141125426	D397Y	0.96008	1.06
rs149792489	A96G	0.92689	1.50	rs147208835	R125W	0.96213	0.89
rs116675104	R167W	0.93715	1.16	rs147681552	P290L	0.92174	1.25
rs121908250	C75S	0.96248	0.90	rs148498685	P87S	0.93523	1.14
rs121908251	C102S	0.96432	1.22	rs149531418	G330E	0.94082	1.12

## Data Availability

The data used in the article are given with the information from where the data were taken, e.g., (http://www.ncbi.nlm.nih.gov/snp/).

## Supplementary Materials

Supplementary Materials
*Supplementary File 1*. Table 1: prediction of phosphorylation sites by NetPhos 3.1 and GPS 3.0. Table 2: CCBE1 ubiquitination prediction results by BDM-PUB. *Supplementary File 2*. Table 1: NetOGlyc 4.0 results for CCBE1 (wild type and final selected mutants). *Supplementary File 3*. Table 1: residue at ligand-binding sites of CCBE1 protein. *Supplementary File 4*. Figure 1: overall significance of the predication tools used in the study (shows the significance of the different predication tools used in the study). Table 1: confirmation of the deleterious nsSNPs by other prediction software (shows the results of the other than SIFT and PolyPhen2 predication tools).
