# Supplementary material for: Predicting the Most Deleterious Missense Nonsynonymous Single-Nucleotide Polymorphisms of Hennekam Syndrome-Causing CCBE1 Gene, In Silico Analysis
Source: ScientificWorldJournal. 2021 Jun 10;2021:6642626. doi: 10.1155/2021/6642626 (PMC8211529; doi:10.1155/2021/6642626)
Supplement: Supplementary Materials — Supplementary File 1. Table 1: prediction of phosphorylation sites by NetPhos 3.1 and GPS 3.0. Table 2: CCBE1 ubiquitination prediction results by BDM-PUB. Supplementary File 2. Table 1: NetOGlyc 4.0 results for CCBE1 (wild type and final selected mutants). Supplementary File 3. Table 1: residue at ligand-binding sites of CCBE1 protein. Supplementary File 4. Figure 1: overall significance of the predication tools used in the study (shows the significance of the different predication tools used in the study). Table 1: confirmation of the deleterious nsSNPs by other prediction software (shows the results of the other than SIFT and PolyPhen2 predication tools). [file 6642626.f1.zip › 6642626.f1/Supplementary Description.docx]

**Supplementary Description:**

**Supplementary File 1:** Table 1: Prediction of Phosphorylation Sites by NetPhos 3.1 and GPS 3.0. Table 2: CCBE1 Ubiquitination Prediction results by BDM-PUB.
**Supplementary 2:** Table 1: NetOGlyc 4.0 Results for CCBE1 (Wild type and final selected Mutants)

**Supplementary 3: Table S3.** Residue at ligand binding sites of CCBE1 protein

**Supplementary 4:** Figure 1: Overall significance of the predication tools used in the study (Shows the significance of the different predication tools used in the study) Table 1: Confirmation of the deleterious nsSNPs by other prediction software (Show the results of the other than sift and PolyPhn2 predication tools)
