# Supplementary material for: Predicting the Most Deleterious Missense Nonsynonymous Single-Nucleotide Polymorphisms of Hennekam Syndrome-Causing CCBE1 Gene, In Silico Analysis
Source: ScientificWorldJournal. 2021 Jun 10;2021:6642626. doi: 10.1155/2021/6642626 (PMC8211529; doi:10.1155/2021/6642626)
Supplement: Supplementary Materials — Supplementary File 1. Table 1: prediction of phosphorylation sites by NetPhos 3.1 and GPS 3.0. Table 2: CCBE1 ubiquitination prediction results by BDM-PUB. Supplementary File 2. Table 1: NetOGlyc 4.0 results for CCBE1 (wild type and final selected mutants). Supplementary File 3. Table 1: residue at ligand-binding sites of CCBE1 protein. Supplementary File 4. Figure 1: overall significance of the predication tools used in the study (shows the significance of the different predication tools used in the study). Table 1: confirmation of the deleterious nsSNPs by other prediction software (shows the results of the other than SIFT and PolyPhen2 predication tools). [file 6642626.f1.zip › 6642626.f1/Table and Fig S4 (1).docx]

S4 Figure: Overall significance of the predication tools used in the study

S 4 Confirmation of the deleterious nsSNPs by other prediction software with threshold

| **AAS** | **Mutation Assessor** | **PROVEAN** | **FATHMM-MKK** | **PhD-SNP** | **PANTHER** | **SNP-GO** | **SNAP2** | **CADD** | **DANN** | **M-CAP** | **FATHMM** | **VEST3** | **MetaLR** | **LRT** | **Mutation Taster** |
| --- | --- | --- | --- | --- | --- | --- | --- | --- | --- | --- | --- | --- | --- | --- | --- |
| G330E | 3.81 | -4.50 | 0.99429 | \| 0.561 \|  \|  \| \| --- \| --- \| --- \| | 456 | \| 0.689 \|  \|  \| \| --- \| --- \| --- \| | 64 | 31 | 0.865 | 0.507 | 0.923 | 0.869 | 0.955 | 0.258 | 0.932 |
| C102S | 1.975 | -5.04 | 0.99398 | \| 0.844 \|  \|  \| \| --- \| --- \| --- \| | 456 | 0.536 | 92 | 28 | 0.963 | 0.218 | 0.855 | 0.932 | 0.973 | 0.262 | 0.955 |
| C174R | 4.8 | -6.36 | 0.98958 | \| 0.874 \|  \|  \| \| --- \| --- \| --- \| | 456 | \| 0.716 \|  \|  \| \| --- \| --- \| --- \| | 93 | 25 | 0951 | 0.833 | 0.922 | 0.968 | 0961 | 0.165 | 0.962 |
| G107D | 1.895 | -2.71 | 0.99364 | \| 0.827 \|  \|  \| \| --- \| --- \| --- \| | 456 | \| 0.443 \|  \|  \| \| --- \| --- \| --- \| | 73 | 26 | 0.987 | 0.192 | 0.874 | 0.965 | 0.977 | 0.198 | 0.847 |
| R125W | 1.08 | -3.63 | 0.98412 | 0.615 | 456 | 0.248 | 61 | 29 | 0.965 | 0.121 | 0.923 | 0.222 | 0.925 | 0.326 | 0.823 |
| G327R | 3.955 | -6.00 | 0.99430 | \| 0.439 \|  \|  \| \| --- \| --- \| --- \| | 456 | \| 0.332 \|  \|  \| \| --- \| --- \| --- \| | 24 | 28 | 0.943 | 0.626 | 0.742 | 0.632 | 0.933 | 0.175 | 0.842 |
| P290L | 2.795 | -4.30 | 0.98986 | \| 0.238 \|  \|  \| \| --- \| --- \| --- \| | 456 | 0.273 | 45 | 29 | 0.810 | 0.397 | 0.723 | 0.589 | 0.910 | 0.196 | 0.823 |
| K355T | 2.075 | -2.04 | 0.98913 | \| 0.616 \|  \|  \| \| --- \| --- \| --- \| | 324 | \| 0.128 \|  \|  \| \| --- \| --- \| --- \| | 40 | 27 | 0.896 | 0.087 | 0.873 | 0.896 | 0.886 | 0.263 | 0.973 |
| Q353R | 2.24 | -2.02 | 0.99310 | \| 0.750 \|  \|  \| \| --- \| --- \| --- \| | 456 | \| 0.264 \|  \|  \| \| --- \| --- \| --- \| | 10 | 27 | 0.835 | 0.274 | 0.843 | 0.986 | 0.935 | 0.023 | 0.943 |
| D336N | 2.24 | -2.15 | 0.99310 | \| 0.520 \|  \|  \| \| --- \| --- \| --- \| | 456 | \| 0.188 \|  \|  \| \| --- \| --- \| --- \| | 21 | 27 | 0.873 | 0.162 | 0.734 | 0.123 | 0.973 | 0.123 | 0.834 |
| T153N | 2.735 | -2.44 | 0.99254 | \| 0.645 \|  \|  \| \| --- \| --- \| --- \| | 455 | \| 0.292 \|  \|  \| \| --- \| --- \| --- \| | 17 | 27 | 0.976 | 0.251 | 0.655 | 0.145 | 0.876 | 0.101 | 0.755 |
| C75S | 1.79 | -3.79 | 0.98250 | \| 0.462 \|  \|  \| \| --- \| --- \| --- \| | 456 | \| 0.206 \|  \|  \| \| --- \| --- \| --- \| | 82 | 27 | 0.147 | 0.129 | 0.552 | 0.589 | 0.847 | 0.236 | 0.652 |
| P87S | 1.7 | -1.94 | 0.98524 | \| 0.534 \|  \|  \| \| --- \| --- \| --- \| | 456 | \| 0.191 \|  \|  \| \| --- \| --- \| --- \| | 04 | 25 | 0.735 | 0.074 | 0.652 | 0.865 | 0.935 | 0.142 | 0.752 |
| T144M | 0.785 | -2.23 | 0.98524 | \| 0.209 \|  \|  \| \| --- \| --- \| --- \| | 361 | \| 0.144 \|  \|  \| \| --- \| --- \| --- \| | 54 | 27 | 0.917 | 0.262 | 0.723 | 0.563 | 0.817 | 0.123 | 0.823 |
| R118L | 1.895 | -2.69 | 0.98628 | 0.754 | 456 | 0.387 | 50 | 23 | 0.853 | 0.308 | 0.242 | 0.654 | 0.753 | 0.012 | 0.642 |
| D397Y | 2.015 | -2.72 | 0.98318 | 0.695 | 361 | 0.134 | 44 | 21 | 0.243 | 0.080 | 0.676 | 0.365 | 0.143 | 0.001 | 0.576 |
| R301W | 2.74 | -3.23 | 0.97946 | \| 0.301 \|  \|  \| \| --- \| --- \| --- \| | 455 | 0.269 | 53 | 27 | 0.796 | 0.085 | 0.332 | 0.985 | 0.696 | 0.123 | 0.732 |
| P249S | 2.805 | -1.98 | 0.98888 | \| 0.111 \|  \|  \| \| --- \| --- \| --- \| | 911 | \| 0.100 \|  \|  \| \| --- \| --- \| --- \| | -1 | 27 | 0.653 | 0.094 | 0.612 | 0.222 | 0.653 | 0.114 | 0.612 |
| D41E | 1.245 | -1.19 | 0.89116 | \| 0.508 \|  \|  \| \| --- \| --- \| --- \| | 220 | \| 0.060 \|  \|  \| \| --- \| --- \| --- \| | -20 | 23 | 0.212 | 0.109 | 0.241 | 0.133 | 0.123 | 0.178 | 0.214 |
| S19N | 1.245 | -0.43 | 0.96461 | 0.463 | 220 | 0.118 | -46 | 24 | 0.654 | 0.158 | 0.536 | 0.162 | 0.554 | 0.012 | 0.136 |
| R167W | 1.275 | -0.20 | 0.99207 | 0.465 | 06 | 0.105 | 46 | 26 | 0.984 | 0.060 | 0.519 | 0.013 | 0.684 | 0.001 | 0.619 |
| A96G | 1.445 | -1.15 | 0.99410 | \| 0.044 \|  \|  \| \| --- \| --- \| --- \| | 456 | 0.021 | -71 | 25 | 0.536 | 0.081 | 0.319 | 0.698 | 0.236 | 0.111 | 0.599 |
| P181S | 1.32 | -0.72 | 0.98462 | \| 0.112 \|  \|  \| \| --- \| --- \| --- \| | 176 | \| 0.105 \|  \|  \| \| --- \| --- \| --- \| | -29 | 21 | 0.112 | 0.037 | 0.173 | 0.593 | 0.212 | 0.001 | 0.673 |

Threshold: Mutation Taster: <0.5 CADD: > 15 MetaLR: > 0.5 M-Cap: > 0.025 PANTHER: probably damaging time > 450my possibly damaging" (450my > time > 200my, "probably benign" (time < 200my). VEST3: > 0.5 LRT: >0.001 PROVEAN: > -2.667 FATHMM-MKK/: < 0.5 PhDSNP: >0.5 SNP-GO: >0.5 SNAP2: −100 (fully neutral) +100 (strong effect) DANN: >0.5 Mutation Assessor: > 0.65 (‐5.545 to 5.975 (higher score ‐> more damaging)) FATHMM: > 0.453, PON-P2: >0.5
