# Supplementary material for: Predicting the Most Deleterious Missense Nonsynonymous Single-Nucleotide Polymorphisms of Hennekam Syndrome-Causing CCBE1 Gene, In Silico Analysis
Source: ScientificWorldJournal. 2021 Jun 10;2021:6642626. doi: 10.1155/2021/6642626 (PMC8211529; doi:10.1155/2021/6642626)
Supplement: Supplementary Materials — Supplementary File 1. Table 1: prediction of phosphorylation sites by NetPhos 3.1 and GPS 3.0. Table 2: CCBE1 ubiquitination prediction results by BDM-PUB. Supplementary File 2. Table 1: NetOGlyc 4.0 results for CCBE1 (wild type and final selected mutants). Supplementary File 3. Table 1: residue at ligand-binding sites of CCBE1 protein. Supplementary File 4. Figure 1: overall significance of the predication tools used in the study (shows the significance of the different predication tools used in the study). Table 1: confirmation of the deleterious nsSNPs by other prediction software (shows the results of the other than SIFT and PolyPhen2 predication tools). [file 6642626.f1.zip › 6642626.f1/Table S1 (1).docx]

**S1 Table:** Prediction of Phosphorylation Sites by NetPhos 3.1 and GPS 3.0

|  | **NetPhos 3.1** | | | **GPS 3.0** | | | |
| --- | --- | --- | --- | --- | --- | --- | --- |
|  | **Position** | **Score** | **Kinase** | **Position** | **Score** | **Cutoff** | **Kinase** |
| Serine (S) | 7 | 0.501 | GSK3 | 7* | 4.959 | 2.819 | AGC/DMPK/GEK/DMPK |
|  | 19 | 0.746 | PKA | 19* | 3.999 | 2.819 | AGC/DMPK/GEK/DMPK |
|  | 48 | 0.473 | CKII | 260* | 4.599 | 4.14 | AGC/PDK1/PDPK1 |
|  | 50 | 0.701 | PKC | 266* | 6.398 | 4.14 | AGC/PDK1/PDPK1 |
|  | 62 | 0.547 | unsp | 284* | 5.185 | 4.14 | AGC/PDK1/PDPK1 |
|  | 63 | 0.840 | unsp | 291* | 4.254 | 4.14 | AGC/PDK1/PDPK1 |
|  | 140 | 0.462 | Cdc2 | 295* | 4.223 | 4.14 | AGC/PDK1/PDPK1 |
|  | 141 | 0.462 | GSK3 | 328* | 6.158 | 4.14 | AGC/PDK1/PDPK1 |
|  | 156 | 0.956 | unsp | 334* | 5.785 | 4.14 | AGC/PDK1/PDPK1 |
|  | 189 | 0.483 | CKI | 316* | 0.003 | 0.001 | AGC/NDR/LATS/LATS1 |
|  | 241 | 0.618 | PKA | 363* | 0.003 | 0.001 | AGC/NDR/LATS/LATS1 |
|  | 260 | 0.582 | Cdk5 | 364* | 0.002 | 0.001 | AGC/NDR/LATS/LATS1 |
|  | 266 | 0.666 | Cdk5 |  |  |  |  |
|  | 284 | 0.707 | unsp |  |  |  |  |
|  | 291 | 0.562 | Cdk5 |  |  |  |  |
|  | 295 | 0.880 | unsp |  |  |  |  |
|  | 316 | 0.982 | unsp |  |  |  |  |
|  | 328 | 0.981 | unsp |  |  |  |  |
|  | 334 | 0.480 | GSK3 |  |  |  |  |
|  | 363 | 0.995 | unsp |  |  |  |  |
|  | 364 | 0.945 | unsp |  |  |  |  |
|  | 376 | 0.867 | unsp |  |  |  |  |
|  | 385 | 0.982 | unsp |  |  |  |  |
| Threonine (T) | 32 | 0.455 | GSK3 | 32* | 3.437 | 2.819 | AGC/DMPK/GEK/DMPK |
|  | 34 | 0.948 | unsp | 197* | 2.842 | 2.819 | AGC/DMPK/GEK/DMPK |
|  | 54 | 0.879 | PKC | 211* | 3.505 | 2.819 | AGC/DMPK/GEK/DMPK |
|  | 55 | 0.686 | unsp | 103* | 3.032 | 2.819 | AGC/DMPK/GEK/DMPK |
|  | 67 | 0.505 | unsp | 153* | 0.002 | 0.001 | AGC/NDR/LATS/LATS2 |
|  | 68 | 0.776 | PKC | 395* | 0.003 | 0.001 | AGC/NDR/LATS/LATS1 |
|  | 103 | 0.473 | Cdc2 |  |  |  |  |
|  | 112 | 0.496 | Cdc2 |  |  |  |  |
|  | 114 | 0.648 | PKC |  |  |  |  |
|  | 153 | 0.483 | DNAPK |  |  |  |  |
|  | 173 | 0.451 | CKI |  |  |  |  |
|  | 175 | 0.560 | PKC |  |  |  |  |
|  | 184 | 0.863 | unsp |  |  |  |  |
|  | 197 | 0.651 | PKC |  |  |  |  |
|  | 201 | 0.614 | PKC |  |  |  |  |
|  | 211 | 0.790 | PKC |  |  |  |  |
|  | 234 | 0.453 | CdC2 |  |  |  |  |
|  | 243 | 0.494 | CdC2 |  |  |  |  |
|  | 351 | 0.529 | CKII |  |  |  |  |
|  | 361 | 0.463 | GSK3 |  |  |  |  |
|  | 393 | 0.888 | unsp |  |  |  |  |
|  | 395 | 0.885 | unsp |  |  |  |  |
| Tyrosine (Y) | 35 | 0.437 | INSR |  |  |  |  |
|  | 57 | 0.531 | unsp |  |  |  |  |
|  | 70 | 0.435 | INSR |  |  |  |  |
|  | 78 | 0.549 | unsp |  |  |  |  |
|  | 90 | 0.606 | unsp |  |  |  |  |
|  | 114 | 0.370 | INSR |  |  |  |  |
|  | 117 | 0.394 | EGFR |  |  |  |  |
|  | 119 | 0.393 | INSR |  |  |  |  |
|  | 131 | 0.450 | INSR |  |  |  |  |
|  | 157 | 0.472 | INSR |  |  |  |  |
|  | 165 | 0.897 | unsp |  |  |  |  |
|  | 180 | 0.941 | unsp |  |  |  |  |
|  | 184 | 0.863 | unsp |  |  |  |  |
|  | 206 | 0.865 | unsp |  |  |  |  |
|  | 232 | 0.492 | unsp |  |  |  |  |
|  | 377 | 0.555 | unsp |  |  |  |  |
|  | 405 | 0.445 | INSR |  |  |  |  |

*Common in both NetPhos 3.1 and GPS 3.0.


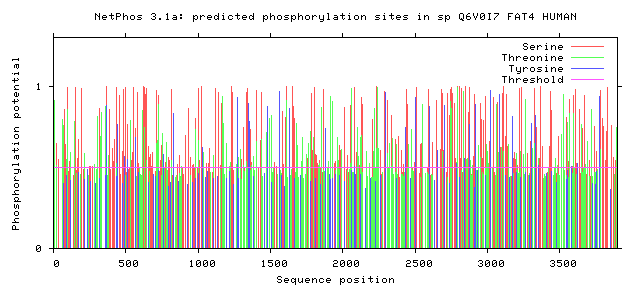


**CCBE1 Ubiquitination Prediction Results by UbPred and BDM-PUB**

| **BDM-PUB** | | |
| --- | --- | --- |
| Position | Score | Threshold |
| 51 | 0.55 | 0.3 |
| 56 | 1.54 | 0.3 |
| 61 | 1.6 | 0.3 |
| 72 | 1.19 | 0.3 |
| 76 | 2.02 | 0.3 |
| 79 | 1.28 | 0.3 |
| 126 | 0.87 | 0.3 |
| 179 | 1.76 | 0.3 |
| 203 | 1.21 | 0.3 |
| 216 | 0.75 | 0.3 |
| 218 | 0.35 | 0.3 |

**Legend for UbPred:**

| Label | Score range | Sensitivity | Specificity |
| --- | --- | --- | --- |
| Low confidence | 0.62 ≤ s ≤ 0.69 | 0.464 | 0.903 |
| Medium confidence | 0.69 ≤ s ≤ 0.84 | 0.346 | 0.950 |
| High confidence | 0.84 ≤ s ≤ 1.00 | 0.197 | 0.989 |
