# Supplementary material for: Predicting the Most Deleterious Missense Nonsynonymous Single-Nucleotide Polymorphisms of Hennekam Syndrome-Causing CCBE1 Gene, In Silico Analysis
Source: ScientificWorldJournal. 2021 Jun 10;2021:6642626. doi: 10.1155/2021/6642626 (PMC8211529; doi:10.1155/2021/6642626)
Supplement: Supplementary Materials — Supplementary File 1. Table 1: prediction of phosphorylation sites by NetPhos 3.1 and GPS 3.0. Table 2: CCBE1 ubiquitination prediction results by BDM-PUB. Supplementary File 2. Table 1: NetOGlyc 4.0 results for CCBE1 (wild type and final selected mutants). Supplementary File 3. Table 1: residue at ligand-binding sites of CCBE1 protein. Supplementary File 4. Figure 1: overall significance of the predication tools used in the study (shows the significance of the different predication tools used in the study). Table 1: confirmation of the deleterious nsSNPs by other prediction software (shows the results of the other than SIFT and PolyPhen2 predication tools). [file 6642626.f1.zip › 6642626.f1/Table S2 (1).docx]

**S2 Table: NetOGlyc 4.0 Results for CCBE1 (Wild type and final selected Mutants)**

| **Position** | **Score** | | | |
| --- | --- | --- | --- | --- |
|  | **Wild type** | **S19N** | **T153N** | **T144M** |
| 7 | 0.835728 | 0.844934 | 0.860456 | 0.822005 |
| 19 | 0.349985 | Loss of Glycosylation site | 0.386128 | 0.356541 |
| 32 | 0.289266 | 0.277823 | 0.328367 | 0.285987 |
| 34 | 0.301072 | 0.282389 | 0.314792 | 0.289733 |
| 48 | 0.390155 | 0.359059 | 0.406118 | 0.373345 |
| 50 | 0.169175 | 0.142759 | 0.148365 | 0.146199 |
| 54 | 0.678348 | 0.632284 | 0.671169 | 0.659976 |
| 55 | 0.424204 | 0.365861 | 0.419966 | 0.403858 |
| 62 | 0.325344 | 0.30039 | 0.302606 | 0.318217 |
| 63 | 0.505812 | 0.45398 | 0.5 | 0.511643 |
| 67 | 0.0416348 | 0.0383557 | 0.0357387 | 0.0461444 |
| 68 | 0.164931 | 0.156867 | 0.152009 | 0.176138 |
| 103 | 0.0875601 | 0.0894442 | 0.0983871 | 0.0728913 |
| 112 | 0.317966 | 0.314895 | 0.341729 | 0.295928 |
| 140 | 0.300427 | 0.308163 | 0.278727 | 0.232767 |
| 141 | 0.137626 | 0.146374 | 0.129779 | 0.0878105 |
| 144 | 0.0438616 | 0.0475192 | 0.039416 | Loss of Glycosylation site |
| 153 | 0.170264 | 0.181044 | Loss of Glycosylation site | 0.121974 |
| 156 | 0.0617472 | 0.0572976 | 0.0364706 | 0.0543887 |
| 173 | 0.15783 | 0.147044 | 0.106164 | 0.126057 |
| 175 | 0.510113 | 0.507553 | 0.449711 | 0.446892 |
| 184 | 0.217757 | 0.173546 | 0.159428 | 0.221649 |
| 189 | 0.312725 | 0.239144 | 0.229824 | 0.310238 |
| 197 | 0.160736 | 0.0897613 | 0.0980154 | 0.150971 |
| 201 | 0.240476 | 0.146969 | 0.13621 | 0.195793 |
| 211 | 0.347105 | 0.261585 | 0.192634 | 0.261188 |
| 234 | 0.31349 | 0.261455 | 0.192029 | 0.300796 |
| 241 | 0.244376 | 0.186495 | 0.171595 | 0.25021 |
| 243 | 0.447955 | 0.341497 | 0.354042 | 0.475285 |
| 260 | 0.812093 | 0.798262 | 0.78215 | 0.845065 |
| 266 | 0.874764 | 0.8534 | 0.822373 | 0.87432 |
| 284 | 0.893382 | 0.865919 | 0.891095 | 0.891019 |
| 291 | 0.923873 | 0.921031 | 0.92499 | 0.916323 |
| 295 | 0.653948 | 0.659676 | 0.659879 | 0.656882 |
| 316 | 0.833678 | 0.809234 | 0.804161 | 0.792984 |
| 328 | 0.91512 | 0.906401 | 0.924434 | 0.894796 |
| 334 | 0.577635 | 0.541888 | 0.610531 | 0.509463 |
| 350 | 0.756063 | 0.753705 | 0.771882 | 0.724664 |
| 361 | 0.899409 | 0.887964 | 0.901095 | 0.895953 |
| 363 | 0.866605 | 0.843474 | 0.859837 | 0.8679 |
| 364 | 0.949935 | 0.94397 | 0.94769 | 0.949607 |
| 376 | 0.852982 | 0.841082 | 0.841891 | 0.828137 |
| 385 | 0.893556 | 0.894966 | 0.882146 | 0.868992 |
| 393 | 0.889859 | 0.894714 | 0.901947 | 0.882575 |
| 395 | 0.851467 | 0.862384 | 0.862691 | 0.838336 |
