# Supplementary material for: Predicting the Most Deleterious Missense Nonsynonymous Single-Nucleotide Polymorphisms of Hennekam Syndrome-Causing CCBE1 Gene, In Silico Analysis
Source: ScientificWorldJournal. 2021 Jun 10;2021:6642626. doi: 10.1155/2021/6642626 (PMC8211529; doi:10.1155/2021/6642626)
Supplement: Supplementary Materials — Supplementary File 1. Table 1: prediction of phosphorylation sites by NetPhos 3.1 and GPS 3.0. Table 2: CCBE1 ubiquitination prediction results by BDM-PUB. Supplementary File 2. Table 1: NetOGlyc 4.0 results for CCBE1 (wild type and final selected mutants). Supplementary File 3. Table 1: residue at ligand-binding sites of CCBE1 protein. Supplementary File 4. Figure 1: overall significance of the predication tools used in the study (shows the significance of the different predication tools used in the study). Table 1: confirmation of the deleterious nsSNPs by other prediction software (shows the results of the other than SIFT and PolyPhen2 predication tools). [file 6642626.f1.zip › 6642626.f1/Table S3 (1).docx]

**Table S3.** Residue at ligand binding sites of CCBE1 protein

| **Site 1** | **Site 2** | **Site 3** |
| --- | --- | --- |
| TYP X 91 | CYS X 113 | VAL X 92 |
| ASP X 91 | TYR X 114 | CYS X 93 |
| VAL X 92 | PRO X 115 | ALA X 94 |
| CYS X 93 | GLY X 116 | GLU X 95 |
| ALA X 94 | TYR X 117 | CYS X 98 |
| GLU X 95 | ARG X 118 |  |
| TYR X 114 | ILE X 135 |  |
| PRO X 115 |  |  |
| TYR X 117 |  |  |
| THR X 153 |  |  |
| LEU X 154 |  |  |
| GLY X 155 |  |  |
| SER X 156 |  |  |
| ILE X 166 |  |  |
| ARG X 167 |  |  |
| ASP X 169 |  |  |
